# Supplementary material for: Phenotype and Treatment Options for Mesenteric Lymph Node Cavitating Syndrome in Coeliac Disease: A Case Series and Literature Review
Source: J Clin Med. 2024 Sep 12;13(18):5417. doi: 10.3390/jcm13185417 (PMC11432660; doi:10.3390/jcm13185417)
Supplement: Supplementary file 1 [file jcm-13-05417-s001.zip › jcm-3144372-supplementary.pdf]

Journal of
Clinical Medicine

MDPI

Table S1. Characteristics of patients with MLNCS including our Centre case series (case 1-6) and those retrieved from the literature review.

References	Demographics			Clinical features	
	Sex	Age of MLNCS diagnosis	MLNCS inaugural (or after CD diagnosis - years)	GI symptoms	Weight loss
**Case 1**	F	45	No (2)	Yes	No
**Case 2**	F	70	No (3)	Yes	Yes
**Case 3**	M	49	Yes	Yes	Yes
**Case 4**	F	61	No (9)		Yes
**Case 5**	F	81	No		
**Case 6**	F	42	No	Yes	Yes
**Tinguria 2021**[1]	M	49	Yes	Yes	Yes
**Torres 2020**[2]	F	47	Yes	Yes	Yes
**Y. Ruch 2019**[3]	M	63	Yes	Yes	Yes
	M	46	Yes	Yes	Yes
	F	47	Yes	Yes	Yes
	F	50	Yes	Yes	Yes
**Ruiz-Cavijo 2017**[4]	F	52	Yes	Yes	Yes
**Forrest 2015**[5]	F	59	Yes	Yes	Yes
**Schwock 2015**[6]	F	71	Yes	Yes	Yes
**Pojoga 2013**[7]	M	51	Yes	Yes	Yes
**Wronski 2012**[8]	M	62	Yes	Yes	Yes
**Ustaoglu 2012**[9]	F	33	Yes	Yes	
**Rodriguez-Sanchez 2012**[10]	M	65	No (10)	Yes	Yes
**Vibhuti 2010**[11]	M	54	No (3)	Yes	Yes
**Sanson 2010**[12]	M	64	No (2)	Yes	Yes
**McBride 2010**[13]	F	71	Yes	No	Yes
**Keer 2010**[14]	M	51		No	
**De Vries 2008**[15]	F	73		Yes	Yes

**Huppert 2004**[16]	M	61	Yes	Yes	Yes
**Huppert 2003**[17]	F	59	No (6)	Yes	Yes
**Schmitz 2002**[18]	F	62	No (17)	Yes	Yes
**Reddy 2002**[19]	F	83	Yes	Yes	Yes
**Arotçarena 2002**[20]	F	39	Yes	Yes	Yes
**Bardella 1999**[21]	F	67	Yes	Yes	Yes
**Bahlouli 1998**[22]	F	42	Yes		Yes
**Howat 1995**[23]	F	50	No (15)		Yes
	M	68	No (1)	Yes	Yes
	M	53	Yes		Yes
	F	44	Yes	Yes	Yes
	M	69	Yes	Yes	Yes
**Burrel 1994**[24]	M	39	Yes	Yes	
**Bulger 1988**[25]	M	53	No (28)	Yes	Yes
**Holmes 1986**[26]	M	38	No (29)	Yes	Yes
**Freeman 1986**[27]	M	70	No (1)	Yes	Yes
**Matuchansky 1984**[28]	F	53	Yes	Yes	Yes
	F	63	Yes	Yes	Yes
	F	30	No (1)	Yes	Yes
	F	26	Yes	Yes	Yes
	M	43	Yes	Yes	Yes
	M	22	No (1)	Yes	Yes
**Hoang 1983**[29]	F	41			
**Marche 1974**[30]	F	38			
**Jones e Gleeson**	M	58			
**Hemet 1969** [31]	M	42			
**Hemet**	F	25			

**References**	**Biology**				**Imaging results**
	**Anemia**	**Howell-Jolly Body**	**Hematinic deficiency**	**Immunology**	
**Case 1**				Positive	Necrotic Lymphadenopathy, Spleen atrophy, calcification
**Case 2**	Yes			Positive	Necrotic Lymphadenopathy, Spleen atrophy
**Case 3**	Yes		Yes	Positive	Necrotic Lymphadenopathy, Spleen atrophy
**Case 4**					Necrotic Lymphadenopathy, Necrotic cancerous mass, Spleen atrophy
**Case 5**				Positive	Necrotic Lymphadenopathy, Spleen atrophy, Calcification
**Case 6**			Yes	Negative	Necrotic Lymphadenopathy, Spleen atrophy
**Tinguria 2021**[1]	Yes		Yes	Positive	Necrotic Lymphadenopathy, Spleen atrophy
**Torres 2020**					Necrotic Lymphadenopathy, Spleen atrophy, Calcification
**Y. Ruch 2019**[3]	Yes	Yes	Yes	Positive	Necrotic Lymphadenopathy, Necrotic cancerous mass, Spleen atrophy, Calcification
	Yes	No	Yes	Positive	Necrotic Lymphadenopathy, Spleen atrophy
	Yes	No	Yes	Positive	Necrotic Lymphadenopathy
	Yes		No		Necrotic Lymphadenopathy, Spleen atrophy
**Ruiz-Cavijo 2017**[4]				Positive	Necrotic Lymphadenopathy
**Forrest 2015**[5]		Yes		Positive	Necrotic Lymphadenopathy, Necrotic cancerous mass, Spleen atrophy, Calcification
**Schwock 2015**[6]		Yes		Positive	Necrotic Lymphadenopathy

**Pojoga 2013**[7]	Yes	Yes	Yes	Positive	Necrotic Lymphadenopathy, Spleen atrophy
**Wronski 2012**[8]	Yes				Necrotic Lymphadenopathy, Spleen atrophy
**Ustaoglu 2012**[9]	Yes		Yes	Positive	Necrotic Lymphadenopathy
**Rodriguez-Sanchez 2012**[10]			Yes	Positive	Necrotic Lymphadenopathy
**Vibhuti 2010**[11]	Yes		Yes	Positive	Necrotic Lymphadenopathy
**Sanson 2010**[12]		Yes		Positive	Necrotic Lymphadenopathy, Spleen atrophy
**McBride 2010**[13]				Positive	Necrotic Lymphadenopathy, Necrotic cancerous mass
**Keer 2010**[14]					Necrotic Lymphadenopathy, Necrotic cancerous mass, Spleen atrophy
**De Vries 2008**[15]					Necrotic Lymphadenopathy, Necrotic cancerous mass, Spleen atrophy
**Huppert 2004**[16]		Yes		Positive	Necrotic Lymphadenopathy
**Huppert 2003**[17]		Yes		Positive	Necrotic Lymphadenopathy, Spleen atrophy
**Schmitz 2002**[18]			Yes	Negative	Necrotic cancerous mass, Spleen atrophy
**Reddy 2002**[19]					Necrotic Lymphadenopathy, Spleen atrophy
**Arotçarena 2002**[20]	Yes	Yes	Yes	Positive	Necrotic Lymphadenopathy, Necrotic cancerous mass, Spleen atrophy
**Bardella 1999**[21]	Yes			Positive	Necrotic Lymphadenopathy
**Bahlouli 1998**[22]		Yes		Positive	Necrotic Lymphadenopathy, Spleen atrophy
**Howat 1995**[23]					Necrotic Lymphadenopathy, Spleen atrophy
					Necrotic Lymphadenopathy, Spleen atrophy

	Yes	Yes			Necrotic Lymphadenopathy, Necrotic cancerous mass
		Yes			Necrotic Lymphadenopathy, Spleen atrophy
		Yes			Necrotic Lymphadenopathy, Necrotic cancerous mass, Spleen atrophy
**Burrel 1994**[24]	Yes	Yes	Yes		Necrotic Lymphadenopathy, Spleen atrophy
**Bulger 1988**[25]		Yes			Necrotic Lymphadenopathy, Necrotic cancerous mass, Spleen atrophy
**Holmes 1986**[26]	Yes	Yes	Yes		Necrotic Lymphadenopathy, Necrotic cancerous mass, Spleen atrophy
**Freeman 1986**[27]	Yes	Yes	No		Necrotic Lymphadenopathy, Spleen atrophy
**Matuchansky 1984**[28]		Yes			Necrotic Lymphadenopathy, Spleen atrophy
		Yes		Positive	Necrotic Lymphadenopathy, Spleen atrophy
		Yes			Necrotic Lymphadenopathy, Spleen atrophy
		Yes			Necrotic Lymphadenopathy, Spleen atrophy
		Yes			Necrotic Lymphadenopathy, Spleen atrophy
		Yes			Necrotic Lymphadenopathy, Spleen atrophy
**Hoang 1983**[29]					Spleen atrophy
**Marche 1974**[30]					Spleen atrophy
**Jones e Gleeson**					Spleen atrophy
**Hemet 1969** [31]					Spleen atrophy
**Hemet**					Spleen atrophy

**References**	**Histology**		**Complications**	**Follow-up**			

	**Villous atrophy**	**Lymphocytic infiltrate**		**Evolution with GFD (improvement)**	**Steroid**	**Death (Years)**	**Cause of death**
**Caso 1**	Yes			Partial	No	No	
**Caso 2**	Yes	Yes	RCD	Marked	Yes	No	
**Caso 3**	Yes	Yes	RCD	Partial	Yes	Yes (9)	COPD
**Caso 4**	Yes	Yes	Lymphoma, RCD	Absent	Yes	Yes (5)	Lymphoma
**Caso 5**	Yes			Absent	Yes	Yes	Cachexia
**Caso 6**	Yes	Yes		Marked	No	No	
**Tinguria 2021**[1]	Yes	Yes		Total	Yes	No	
**Torres 2020**							
**Y. Ruch 2019**[3]	Yes	Yes		Absent	No	Yes (<1)	Complication of MLNCS
	Yes	Yes	Lymphoma, RCD	Absent	Yes	Yes (<1)	Lymphoma
	Yes	Yes		Absent	Yes	Yes (<1)	Cachexia
	Yes	Yes		Absent	No	Yes (>1)	
**Ruiz-Cavijo 2017**[4]	Yes			Partial		No	
**Forrest 2015**[5]		Yes		Total		No	
**Schwock 2015**[6]		Yes	Lymphoma			No	
**Pojoga 2013**[7]	Yes	Yes		Absent		Yes (<1)	Liver failure
**Wronski 2012**[8]	No	Yes	Lymphoma	Absent		Yes (<1)	Lymphoma
**Ustaoglu 2012**[9]	Yes			Partial		No	

**Rodriguez-Sanchez 2012**[10]	Yes	Yes		Partial	Yes	No	
**Vibhuti 2010**[11]	Yes	Yes	RCD				
**Sanson 2010**[12]	Yes	Yes	Lymphoma, RCD	Partial	Yes	Yes (>1)	Septic shock
**McBride 2010**[13]		Yes	Lymphoma	Partial		No	
**Keer 2010**[14]							
**De Vries 2008**[15]	Yes	Yes	Lymphoma, RCD	Absent		Yes (1)	Sepsis
**Huppert 2004**[16]	Yes	Yes	RCD	Partial		No	
**Huppert 2003**[17]	Yes	Yes	RCD		No		
**Schmitz 2002**[18]	Yes	Yes		Partial		No	
**Reddy 2002**[19]	Yes			Partial		No	
**Arotçarena 2002**[20]	Yes	Yes		Partial	No	No	
**Bardella 1999**[21]	Yes	Yes		Total	Yes	No	
**Bahlouli 1998**[22]	Yes			Total		No	
**Howat 1995**[23]	Yes			Absent	No	Yes (>1)	Cachexia
	Yes			Absent	No	Yes (<1)	Sepsis
	Yes			Partial	Yes	No	
	Yes			Partial	Yes	No	
	Yes			Absent	Yes	Yes (>1)	Cachexia
**Burrel 1994**[24]	Yes			Absent	Yes	No	
**Bulger 1988**[25]	Yes	Yes	Lymphoma		No		

**Holmes 1986**[26]	Yes	Yes		Partial	Yes	No	
**Freeman 1986**[27]	Yes		Lymphoma, RCD	Absent	Yes	Yes (>1)	Lymphoma
**Matuchansky 1984**[28]	Yes			Partial	Yes	Yes (>1)	Pneumopathy
	Yes			Total	No	No	
	Yes			Partial	Yes	Yes (>1)	Infectious pericarditis
	Yes			Partial	Yes	Yes (<1)	Cachexia
	Yes			Partial	Yes	Yes (>1)	Cachexia
	Yes			Total	No	No	
**Hoang 1983**[29]	Yes			Total	Yes	No	
**Marche 1974**[30]	Yes			Partial	Yes	Yes	
**Jones e Gleeson**	Yes			Total	Yes	No	
**Hemet 1969** [31]	Yes			Partial	Yes	Yes	
**Hemet**	Yes			Partial	Yes	Yes	

Abbreviation MLNCS: mesenteric lymph node cavitation syndrome, CD: celiac disease, GI: gastrointestinal, RCD: refractory celiac disease, GFD: gluten free diet, COPD: chronic obstructive pulmonary disease.

References

(1) Tinguria, M.; Liaconis, H. Cavitating Mesenteric Lymph Node Syndrome: A Rare Complication of Celiac Disease. A Case Report with Clinicopathologic Features and Review of Literature. *Hum. Pathol. Rep.* **2021**, *26*, 300580. https://doi.org/10.1016/j.hpr.2021.300580.

(2) Torres, L.; Tavares, E. N.; Ghezzi, C. L. A. Calcified Cavitating Mesenteric Lymph Node Syndrome: A Rare Complication of Celiac Disease. *Rev. Esp. Enferm. Dig.* **2021**, *113* (4),

294–295. https://doi.org/10.17235/reed.2020.7494/2020.
(3) Ruch, Y.; Labidi, A.; Martin, A.; Weingertner, N.; Hansmann, Y.; Lefebvre, N.; Andres, E.; Argemi, X.; Dieudonné, Y. [Mesenteric lymph node cavitation in celiac disease: Report of four cases and literature review]. *Rev. Med. Interne* **2019**, *40* (8), 536–544. https://doi.org/10.1016/j.revmed.2019.05.010.
(4) Ruiz-Clavijo García, D.; García de Galdiano Fernández, A.; González de la Higuera Carnicer, B.; Rubio-Marco, I.; Mercado Gutiérrez, M. Cavitating Mesenteric Lymph Node Syndrome: An Uncommon Complication of Celiac Disease. *Rev. Gastroenterol. Mex.* **2017**, *82* (4), 351–353. https://doi.org/10.1016/j.rgmx.2016.05.006.
(5) Forrest, C. E.; Masters, V.; Smith, C. L.; Farshid, G. Case Report: Cavitation of Mesenteric Lymph Nodes as the Presenting Feature of Coeliac Disease. *J. Med. Imaging Radiat. Oncol.* **2015**, *59* (4), 471–473. https://doi.org/10.1111/1754-9485.12274.
(6) Schwock, J.; Hyjek, E. M.; Torlakovic, E. E.; Geddie, W. R. Enteropathy-Associated Intestinal T-Cell Lymphoma in Cavitating Mesenteric Lymph Node Syndrome: Fine-Needle Aspiration Contributes to the Diagnosis. *Diagn. Cytopathol.* **2015**, *43* (2), 125–130. https://doi.org/10.1002/dc.23144.
(7) Pojoga, C.; Ciobanu, L.; Badea, A. F.; Boţan, E.; Caraiani, C.; Hagiu, C.; Băciuţ, G.; Badea, R. 2D Ultrasonography and Contrast Enhanced Ultrasound for the Evaluation of Cavitating Mesenteric Lymph Node Syndrome in a Patient with Refractory Celiac Disease and Enteropathy T Cell Lymphoma. *BMC Gastroenterol.* **2013**, *13*, 26. https://doi.org/10.1186/1471-230X-13-26.
(8) Wronski, M.; Slodkowski, M.; Krasnodebski, I. Gastrointestinal: Cavitating Mesenteric Lymph Node Syndrome as Manifestation of Anaplastic Large Cell Lymphoma. *J. Gastroenterol. Hepatol.* **2012**, *27* (2), 412–412. https://doi.org/10.1111/j.1440-1746.2011.06991.x.
(9) Ustaoglu, M.; Bektas, A.; Bakir, T.; Danaci, M. A Rare Manifestation of Celiac Disease: Mesenteric Lymphadenopathy with Cavitation. *Turk. J. Gastroenterol.* **2012**, *23* (6), 817–818. https://doi.org/10.4318/tjg.2012.0449.
(10) *Rodríguez-Sánchez: Cavitating mesenteric lymph... - Google Scholar.* https://scholar.google.com/scholar_lookup?title=Mesenteric%20lymph%20node%20syndrome%20cavitation%3A%20a%20rare%20complication%20of%20celiac%20disease&author=J.%20Rodr%C3%ADguez-S%C3%A1nchez&publication_year=2012&pages=652-654 (accessed 2024-03-27).
(11) Vibhuti, null; Vishal, K.; Gill, A. Cavitatory Mesenteric Lymph Node Syndrome: A Rare Entity. *Indian J. Radiol. Imaging* **2010**, *20* (1), 66–68. https://doi.org/10.4103/0971-3026.59759.
(12) Sanson, E.; Gassler, N.; Trautwein, C.; Wasmuth, H. E. Cavitating Mesenteric Lymph Node Syndrome: A Rare Complication of Refractory Celiac Disease. *Z. Gastroenterol.* **2010**, *48* (9), 1133–1137. https://doi.org/10.1055/s-0028-1109948.
(13) McBride, O. M. B.; Skipworth, R. J. E.; Leitch, D.; Yalamarthi, S. Cavitating Mesenteric Lymph Node Syndrome in Association with Coeliac Disease and Enteropathy Associated T-Cell Lymphoma: A Case Report and Review of the Literature. *Case Rep. Med.* **2011**, *2010*, e478269. https://doi.org/10.1155/2010/478269.

(14) Keer, D.; Jeon, P.; Borganonkar, M.; Potoczny, S. Calcified Cavitating Mesenteric Lymph Node Syndrome: Case Presentation and Literature Review. *Can. J. Gastroenterol. J. Can. Gastroenterol.* **2010**, *24* (6), 355–358. https://doi.org/10.1155/2010/530475.

(15) de Vries, A. P. J.; Bakker, S. J. L.; Kallenberg, C. G. M.; Gans, R. O. B.; Bongaerts, A. H. H.; Kluin, P. M. Cavitating Lymph Node Syndrome. *Neth. J. Med.* **2008**, *66* (9), 403–404.

(16) Huppert, B. J.; Farrell, M. A.; Kawashima, A.; Murray, J. A. Diagnosis of Cavitating Mesenteric Lymph Node Syndrome in Celiac Disease Using MRI. *AJR Am. J. Roentgenol.* **2004**, *183* (5), 1375–1377. https://doi.org/10.2214/ajr.183.5.1831375.

(17) Huppert, B. J.; Farrell, M. A. Case 60: Cavitating Mesenteric Lymph Node Syndrome. *Radiology* **2003**, *228* (1), 180–184. https://doi.org/10.1148/radiol.2281011429.

(18) Schmitz, F.; Herzig, K.-H.; Stüber, E.; Tiemann, M.; Reinecke-Lüthge, A.; Nitsche, R.; Fölsch, U. R. On the Pathogenesis and Clinical Course of Mesenteric Lymph Node Cavitation and Hyposplenism in Coeliac Disease. *Int. J. Colorectal Dis.* **2002**, *17* (3), 192–198. https://doi.org/10.1007/s00384-001-0361-8.

(19) Reddy, D.; Salomon, C.; Demos, T. C.; Cosar, E. Mesenteric Lymph Node Cavitation in Celiac Disease. *Am. J. Roentgenol.* **2002**, *178* (1), 247–247. https://doi.org/10.2214/ajr.178.1.1780247.

(20) Arotçarena, R.; Hammel, P.; Terris, B.; Guth, A.; Bernades, P.; Ruszniewski, P. [Regression of mesenteric lymph node cavitation syndrome complicating celiac disease after a gluten free diet]. *Gastroenterol. Clin. Biol.* **2000**, *24* (5), 579–581.

(21) Bardella, M. T.; Trovato, C.; Quatrini, M.; Conte, D. Mesenteric Lymph Node Cavitation: A Rare Hallmark of Celiac Disease. *Scand. J. Gastroenterol.* **1999**, *34* (12), 1257–1259. https://doi.org/10.1080/003655299750024805.

(22) Bahlouli, F.; Seror, O.; Mathieu, E.; Fain, O.; Amrane, H.; Ghenassia, C.; Coderc, E.; Sellier, N. [Mesenteric lymph node cavitation disclosing celiac disease in adults]. *J. Radiol.* **1998**, *79* (5), 431–433.

(23) Howat, A. J.; McPhie, J. L.; Smith, D. A.; Aqel, N. M.; Taylor, A. K.; Cairns, S. A.; Thomas, W. E.; Underwood, J. C. Cavitation of Mesenteric Lymph Nodes: A Rare Complication of Coeliac Disease, Associated with a Poor Outcome. *Histopathology* **1995**, *27* (4), 349–354. https://doi.org/10.1111/j.1365-2559.1995.tb01525.x.

(24) Burrell, H. C.; Trescoli, C.; Chow, K.; Ward, M. J. Case Report: Mesenteric Lymph Node Cavitation, an Unusual Complication of Coeliac Disease. *Br. J. Radiol.* **1994**, *67* (803), 1139–1140. https://doi.org/10.1259/0007-1285-67-803-1139.

(25) Bulger, K.; Griffin, M.; O'Brien, M.; Crowe, J. Lymphoma in the Mesenteric Lymph Node Cavitation Syndrome. *Gastroenterology* **1988**, *94* (2), 553. https://doi.org/10.1016/0016-5085(88)90468-4.

(26) Holmes, G. K. Mesenteric Lymph Node Cavitation in Coeliac Disease. *Gut* **1986**, *27* (6), 728–733. https://doi.org/10.1136/gut.27.6.728.

(27) Freeman, H. J.; Chiu, B. K. Small Bowel Malignant Lymphoma Complicating Celiac Sprue and the Mesenteric Lymph Node Cavitation Syndrome. *Gastroenterology* **1986**, *90* (6), 2008–2012. https://doi.org/10.1016/0016-5085(86)90275-1.

(28) Matuchansky, C.; Colin, R.; Hemet, J.; Touchard, G.; Babin, P.; Eugene, C.; Bergue, A.;

Zeitoun, P.; Barboteau, M. A. Cavitation of Mesenteric Lymph Nodes, Splenic Atrophy, and a Flat Small Intestinal Mucosa. Report of Six Cases. *Gastroenterology* **1984**, *87* (3), 606–614.

(29) Hoang, C.; Galian, A.; Maitre, F.; Degois, T.; Celerier, M.; Modigliani, R. [Total villous atrophy, mesenteric lymph-node cavitation, splenic atrophy. An unusual form of celiac disease in adults, apropos of a new case]. *Ann. Pathol.* **1983**, *3* (3), 251–256.

(30) Marche, C.; Bocquet, L.; Mignon, M.; Preel, J. L. [Malabsorption syndrome with mesenteric lymph node cavitation and splenic atrophy. Apropos of a new anatomo-clinical case]. *Sem. Hopitaux Organe Fonde Par Assoc. Enseign. Med. Hopitaux Paris* **1974**, *50* (13), 879–886.

(31) Hemet, J.; Bourquelot, R.; Colin, R. [Malabsorption and mesenteric cavitation]. *Arch. Anat. Pathol. (Paris)* **1969**, *17* (2), 115–118.
